# Temperature-dependent microbial dynamics in touchless sensor faucets during short-term stagnation

**DOI:** 10.1016/j.ese.2025.100624

**Published:** 2025-09-24

**Authors:** Anran Ren, Zihan Dai, Xiaoming Li, Walter van der Meer, Joan B. Rose, Gang Liu

**Affiliations:** a Jiangsu Key Laboratory of Industrial Pollution Control and Resource Reuse, School of Environmental Engineering, Xuzhou University of Technology, Xuzhou, 221018, China; b Key Laboratory of Drinking Water Science and Technology, Research Centre for Eco-Environmental Sciences, Chinese Academy of Sciences, Beijing, 100085, China; c University of Chinese Academy of Sciences, Beijing, China; d School of Civil and Environmental Engineering, Georgia Institute of Technology, Atlanta, GA, 30332, USA; e Science and Technology, University of Twente, P.O. Box 217, 7500AE Enschede, the Netherlands; f Oasen Drinkwater, PO BOX 122, 2800 AC, Gouda, the Netherlands; g Department of Fisheries and Wildlife, Michigan State University, East Lansing, MI, 48823, USA; h Sanitary engineering, Department of Water Management, Faculty of Civil Engineering and Geosciences, Delft University of Technology, P.O. Box 5048, 2600 GA, Delft, the Netherlands

**Keywords:** Touchless sensor faucet, Short stagnation, Temperature, Microbial water quality, *L. pneumophila*

## Abstract

Microbial contamination in building plumbing systems poses significant risks to public health at the point of use. Stagnation and warm temperatures are well-known drivers of microbial regrowth, but the effects of common short-term stagnation in touchless sensor faucets—widely used for hygiene and comfort—remain poorly understood. Here we show that microbial water quality in touchless sensor faucets changes during short-term stagnation (0.25–10 h) at varying temperatures (10, 30, and 40 °C). We identify two pivotal time points—2 and 4 h—where microbial diversity decreases and *Legionella pneumophila* concentrations increase significantly, driven by accelerated chlorine decay and biofilm contributions. Heating to 30 °C maximizes microbial biomass (measured as ATP) but minimizes *L. pneumophila* proliferation, whereas 40 °C reduces biomass while promoting *L. pneumophila* growth. These findings reveal a temperature-dependent microbial water quality guarantee period of 2–4 h, beyond which flushing is necessary to mitigate health risks. Optimizing faucet temperatures between 30 and 40 °C could balance microbial safety, user comfort, and energy efficiency, offering practical guidance for managing water quality in modern plumbing systems.

## Highlights

• Residual chlorine and specie richness decline while ATP rises during 0–10 h stagnation.
• Microbial water quality exhibits turning points at 2 h and 4 h of stagnation.
• Biofilm contributions dominate after 4 h at 10 °C, but only 2 h at 30/40 °C.
• Sharp microbial shifts occur when residual chlorine falls below 0.3 mg L^−1^.
• 30 °C heating kills *L. pneumophila* better than 40 °C, balancing safety and energy.

## Graphical abstract

**Figure:**
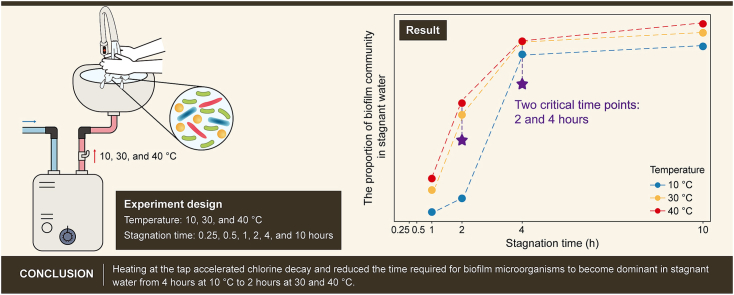

## 1 Introduction

Stagnation and warm temperatures are two major causes of microbial water quality deterioration in building plumbing systems [[1], [2], [3], [4]]. Stagnation in building plumbing systems promotes the growth of microbes, and the growth of opportunistic pathogens has been widely reported after various stagnation times, including overnight (e.g., 10–24 h) [[5], [6], [7]], the weekend (e.g., 2 days) [8], a week (6–7 days) [1,9], and months (e.g., COVID-19 pandemic lockdown) [[10], [11], [12]]. In addition to promoting the growth of opportunistic pathogens (e.g., *Legionella pneumophila*), stagnation can induce the introduction of more microbes from biofilm into stagnant water [13,14]. While substantial attention has been directed toward overnight and extended stagnation periods, the more prevalent short-term stagnation events (≤10 h) [[15], [16], [17]]—which occur routinely in daily life—have received limited attention in the existing literature. Critical questions, such as when significant deterioration occurs and how long the microbial water quality at taps can be maintained, remain unanswered.

Many studies have focused on the impact of temperature on water microbiology in building plumbing systems, particularly in hot water systems. Overnight stagnation can increase water temperature by more than 10 °C (from 4 to 8 to 15–23 °C) [9], which is accompanied by a 5- to 11-fold increase in biomass [18]. Most research has been conducted on hot water systems (e.g., showers), examining temperatures between 39 and 58 °C [3,19,20]. It is believed that raising the temperature to a critical level can control the growth of opportunistic pathogens; for example, maintaining a water temperature above 55 °C reduces the risk of *L. pneumophila* and nontuberculous mycobacteria (NTM) [21,22]. However, the implementation of high-temperature processes undermines energy conservation, and *L. pneumophila* has been detected even at a water temperature of 58 °C due to stagnation [3,19]. Regardless of heating temperature, heated water needs to be mixed with cold water to achieve a comfortable temperature before it can be used by customers, usually between 32 and 43 °C [23]. Although the impact of temperature ranges on microbes has been examined in the literature, the influence of heating temperature (e.g., 30 and 40 °C) on microbial water quality remains underexplored.

Sensor heating faucets have become increasingly popular in the pursuit of comfort and hygiene, especially in cold regions and public buildings. According to a recent report, the global market size is around USD 4 billion (∼80 million devices, 29 % occupancy), and the Chinese market is around.

USD 1 billion (∼20 million devices, 20 % occupancy) [24]. The use of these faucets will be further increased in schools, shopping malls, stations, and other public places due to their touchless feature, which minimizes the risk of contact-based pathogen transmission. However, unlike conventional hot water systems, these faucets lack a mixing mechanism, thereby restricting their use to relatively low temperatures (below 45 °C). Moreover, with sensor-activated faucets, users are inevitably exposed to the initial flush of stagnant water, as the flow ceases when hands are withdrawn. Therefore, for better understanding and management of microbial safety, it is imperative to investigate how stagnation duration and heating temperature influence microbial water quality.

Disinfectant residual is a critical parameter that diminishes over time during stagnation, with its decay rate influenced by temperature [1,25,26]. More importantly, chlorine decay is intrinsically linked to microbial growth potential, as diminishing disinfectant levels create favorable conditions for microbial regrowth [1,27]. The objective of this work was to investigate the dynamics of chlorine decay and bacterial growth at different temperatures (i.e., 10, 30, and 40 °C) using high-resolution sampling (i.e., 0.25, 0.5, 1, 2, 4, and 10 h). Our findings provide valuable guidance for setting optimal stagnation times and heating temperatures to control microbial risks while ensuring comfortable hand washing.

## 2 Materials and methods

### 2.1 Experimental setup and operation

Touchless sensor faucets equipped with 10 L electric water heaters (model 10B2, A.O. Smith) were set up in the laboratory of the Research Center for Eco-Environmental Sciences, Chinese Academy of Sciences. The water heater, sized 350 mm × 280 mm × 350 mm and with 2000 W power, was lined with silicide. The touchless sensor faucet (inner diameter 22 mm) was made of zinc alloy. Touchless sensor faucets and water heaters were connected by 1 m polyvinyl chloride hoses (inner diameter 7 mm). Notably, the faucet system lacked a mixing valve for hot and cold water; the heater's set temperature directly determined the outlet water temperature, which was usually below 45 °C. The system was operated sequentially: following a 10-week acclimation period without heating (∼10 °C), the heater was set to 30 °C for 10 weeks, followed by 40 °C for another 10 weeks [3,28]. The heater was programmed to reheat water when the tank temperature dropped 5 °C below the target. At setpoints of 30 and 40 °C, the water heater started reheating approximately every 4–5 h. Thus, for a stagnation period of 10 h, the system reheated two or three times, whereas shorter stagnation periods typically involved a single reheating cycle.

Traditionally, stagnation time typically refers to the duration of water remaining in the distal pipes (usually at room temperature, regardless of the set point) or the interval between large flushing events in the tank. In contrast, this study defines stagnation time as the period between the collection of fresh and stagnant water samples within the entire touchless sensor faucet system. During this stagnation period, the touchless sensor faucet remained unused, and no fresh water entered the water heater tank. Under experimental conditions, water flowing from the faucet triggered continuous replenishment by the heater, maintaining a constant 10 L tank volume. During the 10 weeks of acclimation time, the touchless sensor faucet was flushed twice a day, each time for 5 min at a flow rate of 9 L min^−1^, immediately followed by sample collection.

### 2.2 Sample collection and DNA extraction

Following the 10-week acclimation period at each temperature setting, fresh water and stagnant water samples were collected. For fresh water (FW) samples, the touchless sensor faucet was flushed for 5 min at a flow rate of approximately 9 L min^−1^—equivalent to 4.5 times the tank volume—until the outlet water temperature stabilized. Then, 2 L of fresh water were collected. Residual chlorine, total chlorine, and water temperature of the fresh water samples were measured immediately. Chlorine concentrations were measured using the standard N,N-Diethyl-p-phenylenediamine method with a DR300 spectrophotometer, while temperature was measured using a digital electronic thermometer.

For stagnant water (SW) samples, 2 L of stagnant water were collected after 0.25, 0.5, 1, 2, 4, and 10 h of stagnation. After each stagnant water sample was collected, the touchless sensor faucet was flushed for 5 min at a flow rate of 9 L min^−1^, effectively replacing 4.5 times the volume of the water tank. Once the outlet temperature stabilized, a corresponding FW sample was collected. For each stagnation interval, a paired FW sample was obtained. All FW and SW samples for a given set—comprising six stagnation times—were collected within a 24-h period, and triplicate sets were obtained at each temperature over three consecutive days. This yielded 18 paired FW and SW samples (i.e., 36 water samples) per temperature condition. In total, 54 FW and 54 SW samples were collected across all temperature conditions. In addition to water samples, triplicate biofilm (BF) samples were collected at each temperature from the internal surfaces of the water heater, connecting hoses, and touchless sensor faucet using sterile rayon swabs (Copan, Brescia, Italy). This resulted in 9 biofilm samples at each temperature and 27 biofilm samples in total. Overall, 108 water samples and 27 biofilm samples were collected.

All samples were processed within 24 h of sampling. Water samples were filtered through 0.2 μm polycarbonate membrane filters (Whatman, United Kingdom), and both filters and biofilm swabs were stored in sterile centrifuge tubes at −20 °C for subsequent deoxyribonucleic acid (DNA) extraction. DNA was extracted using the FastDNA Spin Kit for Soil (MP Biomedicals, Santa Ana, California, United States) according to the manufacturer's instructions.

### 2.3 Adenosine triphosphate measurement

To assess active biomass, we measured intracellular adenosine triphosphate (ATP) concentrations. Prior to sampling, each water container was gently agitated to ensure homogeneity. A 100 mL aliquot was withdrawn using a syringe fitted with a 0.2 μm syringe filter (Promega, United States); the sample was discarded after filtration, with biomass retained on the filter. Next, 2 mL of lysis reagent was introduced into the syringe using a pipette, and the plunger was depressed just enough to wet the filter. The syringe was left undisturbed at room temperature for 10 min. We then positioned the syringe over a sterile 5 mL centrifuge tube, expelled the lysate, and collected the flow-through for analysis. We quantified ATP concentrations using a Glomax luminometer (Promega, United States) in combination with BacTiter-Glo™ reagent, following the manufacturer's instructions [29]. Each sample was assayed in triplicate.

### 2.4 qPCR analysis

Quantitative polymerase chain reaction (qPCR) targeting the *mip* gene of *L. pneumophila* was performed according to the previously published method [30] (Supplementary Table S1). Quantification was carried out using a Quant Gene 9600 Real-Time PCR instrument (Bioer, China). Each 10 μL reaction contained 5 μL of SsoAdvanced Universal Probes Supermix (Bio-Rad), 0.5 μL of each primer (0.25 μM), 0.2 μL of each probe (0.1 μM), 2.8 μL of water, and 1 μL of template DNA. The qPCR protocol consisted of 40 amplification cycles. Standard curves were generated using synthetic oligonucleotides (Supplementary Table S2).

All qPCR runs for the *mip* gene included triplicate standard curves consisting of serial dilutions ranging from 10^1^ to 10^7^ copies μL^−1^, triplicate negative controls, and triplicate test samples. The limit of detection (LOD) and limit of quantification (LOQ) for the qPCR reaction targeting the *mip* gene were 5 and 10 copies per reaction, respectively, as described in prior research [31,32]. Amplification efficiencies, LOQs, LODs, and standard curves are summarized in Supplementary Table S3.

### 2.5 Sequencing and data processing

We applied 16S ribosomal RNA (16S rRNA) gene amplicon sequencing (paired-end 250 bp, Illumina NovaSeq) to microbiome communities in water and biofilm samples. Universal primers 341F (5′-CCTACGGGNGGCWGCAG-3′) and 785R (5′-GACTACHVGGGTATCTAATCC-3′) [29] were used for polymerase chain reaction (PCR) amplification. For biofilm samples at 10 and 30 °C, one sample had no detectable PCR product and was excluded from sequencing. Raw sequences were first processed using Figaro v1.1.2 to determine optimal trimming parameters [33]. Then, DADA2 v1.21.0 was employed for quality filtering and primer removal, error rate learning, sample inference, redundancy removal (duplicate sequences), paired read merging, amplicon sequence variant (ASV) table construction, and chimera removal [34]. Taxonomy classification was performed using the SILVA138.1 database [35], and phylogenetic reconstruction was conducted using QIIME2 2021.11 [36]. Sequencing data are available in the National Center for Biotechnology Information database under accession number PRJNA1167446.

We used the observed and Shannon indices to determine Alpha diversity and evaluated beta diversity through principal coordinates analysis (PCoA) based on weighted UniFrac metrics. Permutational multivariate analysis of variance (PERMANOVA) was used to test and determine the significance of different grouping factors on sample differences (*p* < 0.05) [37]. Linear discriminant analysis effect size (LEfSe) was used to identify ASVs significantly enriched by the selected factors (LDA score >3). The resulting ASV tables were then used for source tracking with SourceTracker [38], which employs a Bayesian framework to compare the community profiles of “source” and “sink” samples and estimate the proportional contribution of each potential source to the defined sinks.

## 3 Results

### 3.1 Changes in residual chlorine and adenosine triphosphate

**Fig. 1:**
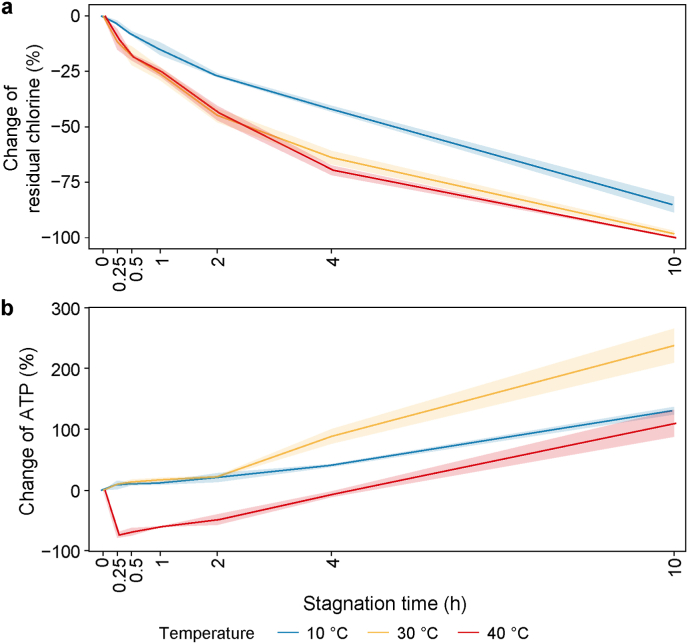
Disinfectant decay (**a**) and microbial growth (**b**) with the increase of stagnation time at different temperatures. Line plots represent mean values with error bands (mean ± standard deviation). ATP: adenosine triphosphate.

The changes in residual chlorine and adenosine triphosphate with stagnation at different temperatures are presented (Fig. 1). The concentrations of residual chlorine in fresh water did not differ significantly between temperatures and were in the range of 0.40–0.52 mg L^−1^. The decay of residual chlorine increased with stagnation time at all temperatures and was sharper at 30 and 40 °C than at 10 °C (Fig. 1a). After 10 h of stagnation, the highest decay rate was observed at 40 °C (0.041 mg L^−1^ h^−1^), followed by 30 °C (0.035 mg L^−1^ h^−1^) and 10 °C (0.028 mg L^−1^ h^−1^). Within short stagnation periods (0.25–10 h), decay rates decreased over time but increased with an increase in temperature. Total chlorine exhibited a similar pattern (Supplementary Fig. S1).

The ATP concentrations increased with stagnation time at all temperatures (Fig. 1b). At 10 and 30 °C, the ATP changes followed exactly the same trend for the first 2 h of stagnation, but at 30 °C they rose sharply between 2 and 4 h, reaching levels after 10 h that were roughly twice those at 10 °C (237 ± 28 % versus 130 ± 6 %). At 40 °C, ATP initially declined sharply at 0.25 h (−73 ± 4 %), suggesting partial microbial inactivation by heating. Thereafter, ATP increased steadily, reaching levels after 10 h (110 ± 22 %) comparable to those at 10 °C.

### 3.2 Changes in diversity and structure of bacterial communities

In total, 5,944,928 raw sequences were obtained for the 133 samples (54 FW, 54 SW, and 25 BF), which were assigned to 12,972 ASVs. The rarefaction curves eventually plateaued, indicating that sufficient sample coverage was achieved in this work (Supplementary Fig. S2). After rarefaction to 4167 reads per sample—the lowest sequencing depth—554,211 sequences were remained, representing 4719 ASVs.

#### 3.2.1 Alpha diversity

**Fig. 2:**
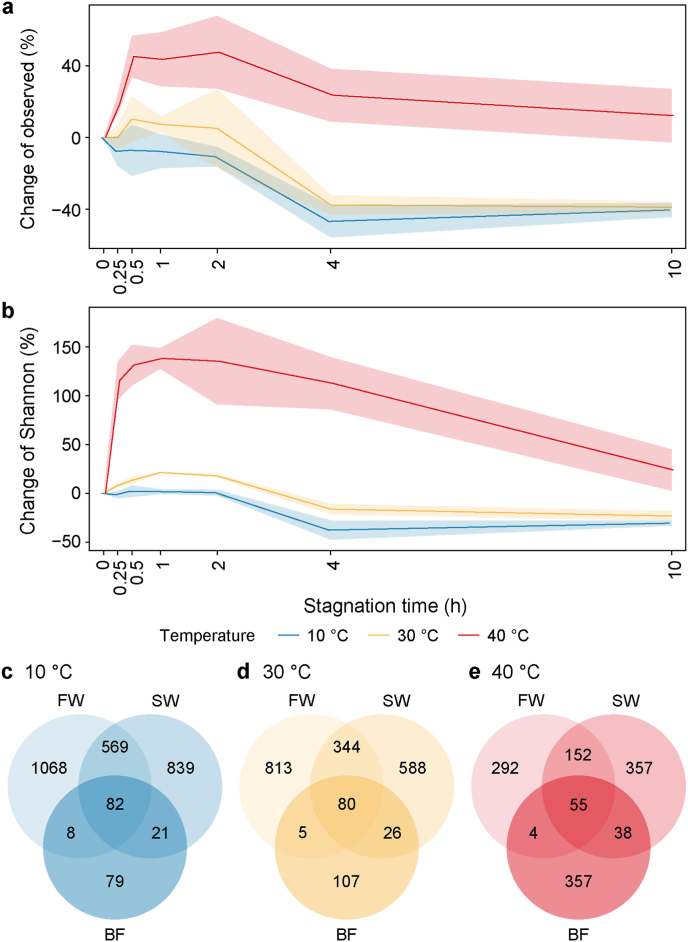
The changes in alpha diversity with stagnation time at different temperatures. **a**, The number of the observed amplicon sequence variant (ASVs). **b**, Shannon index. **c**–**e**, Venn diagram of shared ASVs among fresh water (FW), stagnant water (SW), and biofilm (BF) at 10 °C (**c**), 30 °C (**d**), and 40 °C (**e**). Line plots in panel **a** and panel **b** represent mean values with error bands (mean ± standard deviation).

In the fresh water without any stagnation, 277 ± 8, 209 ± 7, and 101 ± 9 ASVs were observed at 10, 30, and 40 °C, respectively (Supplementary Fig. S3a). For all temperatures, the number of observed ASVs decreased sharply until 4 h and then remained relatively stable until 10 h (Fig. 2a). The initial patterns, however, differed among temperatures. At 10 °C, ASV richness declined continuously with stagnation time, with slow decreases before 2 h and after 4 h, but a marked drop (∼40 %) between 2 h and 4 h. At 30 °C, the trend was similar, except for a slight initial increase at 0.5 h (10.3 %), indicating the release of taxa from biofilm into the water. At 40 °C, a similar initial increase was observed, but longer (2 h) and sharper (47.7 %) than at 30 °C, making the number of observed ASVs higher than in the fresh water at 10 h. This indicates that the biofilm likely contained a greater diversity of species, while the fresh water contained fewer. After stagnation, species loss from fresh water was outweighed by the influx of taxa from biofilms, leading to a higher species count in the stagnant water than in the fresh water. The Shannon index followed the same trend as the number of observed ASVs (Supplementary Fig. S3b; Fig. 2b). The early increases at 30 °C and 40 °C are consistent with the release of biofilm-associated taxa at higher temperatures, an effect not observed at 10 °C. These dynamics—species loss during stagnation coupled with increased biofilm contributions—were further supported by Venn diagram analyses (Fig. 2c–e).

#### 3.2.2 Beta diversity

**Fig. 3:**
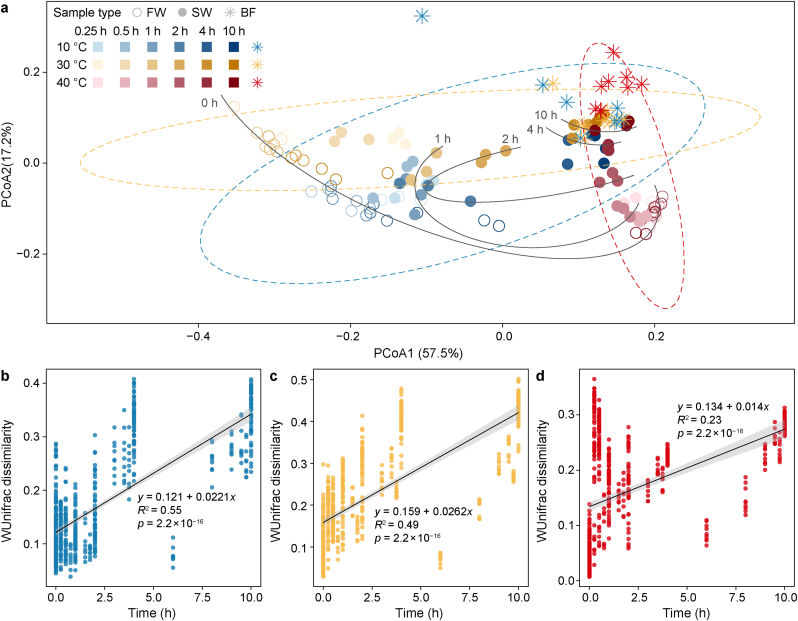
Development of microbial communities based on weighted UniFrac distances. **a**, PCoA plot based on weighted UniFrac dissimilarities for 10, 30, and 40 °C. The dashed ellipses correspond to 95 % confidence regions, the grey lines underscore the stagnation time development (0–10 h, 0 h stands for fresh water). The sample types comprised fresh water (FW), stagnant water (SW), and biofilm (BF). **b**–**d**, Temporal decay in pairwise weighted UniFrac distances at 10 °C (**b**), 30 °C (**c**), and 40 °C (**d**). Regression lines show the linear fits, and the grey-shaded regions indicate 95 % confidence intervals.

The PCoA plot based on the weighted UniFrac metrics revealed a significant effect of temperature on the bacterial community (Adonis, *F* = 34.4, *p* = 0.001; Betadisper, *F* = 1.7, *p* = 0.187) (Fig. 3a). For fresh water, communities differed significantly across temperatures (*p* < 0.001). For stagnant water, temperature-related dissimilarities were most pronounced at shorter stagnation times but diminished with increasing stagnation. When the stagnation time approached 4 h and 10 h, the microbial communities of all temperatures clustered together, resembling the biofilm community and suggesting a dominant contribution of biofilm after 4 h of stagnation. Focusing on time dynamics, all temperature groups showed significant temporal decay, with bacterial communities at each temperature becoming increasingly dissimilar as stagnation time increased (*p* < 0.001) (Fig. 3b–d).

### 3.3 Changes in bacterial community composition

At the phylum level, all water samples were dominated by Proteobacteria (32.4–95.0 %), regardless of temperature and stagnation (Supplementary Fig. S4), followed in descending abundance by Cyanobacteria, Planctomycetota, Actinobacteriota, and Firmicutes. Comparing fresh water with stagnant water (10 h), the relative abundance of Cyanobacteria decreased (19.2 ± 4.6 % to 5.3 ± 3.0 %) at all temperatures, while the relative abundance of Planctomycetota remained stable at 40 °C (2.3 ± 2.7 %) and decreased at 10 °C (12.0 ± 4.6 % to 0.9 ± 0.4 %) and 30 °C (25.2 ± 4.5 % to 0.8 ± 0.2 %). With increasing stagnation time, the relative abundance of Proteobacteria in stagnant water increased from 60.2 ± 5.2 % to 92.4 ± 4.2 %, while Cyanobacteria and Planctomycetota decreased from 22.3 ± 5.6 % to 5.3 ± 3.0 % and from 14.1 ± 7.3 % to 1.6 ± 2.2 %, respectively.

**Fig. 4:**
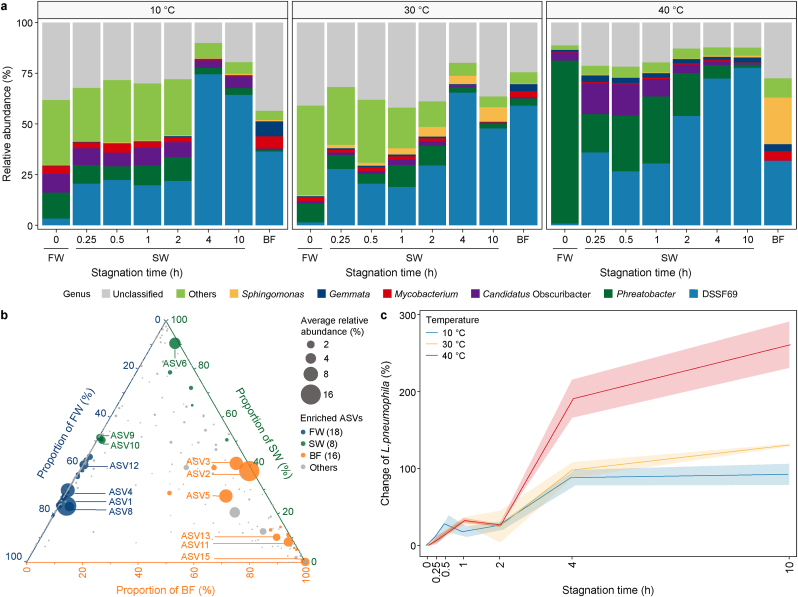
Changes in bacterial community composition with stagnation at different temperatures. **a**, Genus-level community composition of fresh water (FW), stagnant water (SW), and biofilm (BF). Genera with relative abundance lower than 5 % are grouped as “Others.” **b**, Ternary plot depicting the compartment relative abundance of all amplicon sequence variants (ASVs) for FW, SW, and BF. The three vertices represent the three variables, with parenthetical values indicating the number of significantly enriched ASVs for each variable. Each axis corresponds to one variable, uniformly scaled from 0 to 100 %. Each dot corresponds to an ASV; its position represents its relative abundance with respect to each compartment, and its size represents the average relative abundance across all three compartments. Coloured circles represent ASVs enriched in one compartment compared with the others, whereas grey circles represent ASVs that are not significantly enriched in a specific compartment. **c**, The impact of temperature and stagnation time on the changes in gene copy number of *Legionella pneumophila* in stagnant water. Line plots represent mean values with error bands (mean ± standard deviation).

At the genus level, all water samples contained mainly *DSSF69* spp. (Sphingomonadaceae family), followed by *Phreatobacter* spp., *Candidatus* Obscuribacter spp., *Mycobacterium* spp., *Gemmata* spp., and *Sphingomonas* spp. (in descending order) (Fig. 4a). At all temperatures, the relative abundance of *DSSF69* spp. Increased significantly with stagnation (1.8 ± 1.4 % to 40.3 ± 20.9 %, *p* < 0.05), particularly after 4 h, and its relative abundance increased to 66.6 ± 9.8 %. This may have been released from the biofilm, where *DSSF69* spp. was also highly abundant (42.7 ± 12.1 %).

In contrast, *Phreatobacter* spp. was inhibited by stagnation and decreased significantly, particularly at 40 °C, from 79.9 ± 3.0 % to 18.0 ± 11.0 % (*p* < 0.05). The enrichment of *Phreatobacter* spp. In fresh water, *DSSF69* spp. In stagnant water, and biofilm was confirmed by LEfSe analysis (Supplementary Fig. S5 and Table S4). The ASVs enriched in fresh water were absent or rarely present in biofilm, while the ASVs enriched in stagnant water were shared with both fresh water (ASV9, ASV10) and biofilm (ASV10), indicating the possible origins of these species (Fig. 4b).

Temperature and stagnation both strongly influenced *L. pneumophila* gene copy numbers (hereafter “numbers”), with increases observed under all conditions (Fig. 4c). In fresh water, *L. pneumophila* concentrations ranged from (0.6–1.1) × 10^3^, (0.1–0.6) × 10^3^, and (0.4–1.1) × 10^3^ gene copies mL^−1^ at 10, 30, and 40 °C, respectively, with the lowest concentration observed at 30 °C. The number of *L. pneumophila* increased with stagnation time at all temperatures, with the steepest rise occurring between 2 h and 4 h, particularly at 40 °C. After 4 h, the number of *L. pneumophila* remained stable at 10 °C but continued increasing at 30 and 40 °C, though at a much lower rate than that of 2 h–4 h. By 10 h, the number of *L. pneumophila* had increased by 92.4 %, 130.5 %, and 260.8 % at 10, 30, and 40 °C, respectively, suggesting a clear effect of the applied temperature.

### 3.4 Source apportionment of stagnant water microbes by SourceTracker

**Fig. 5:**
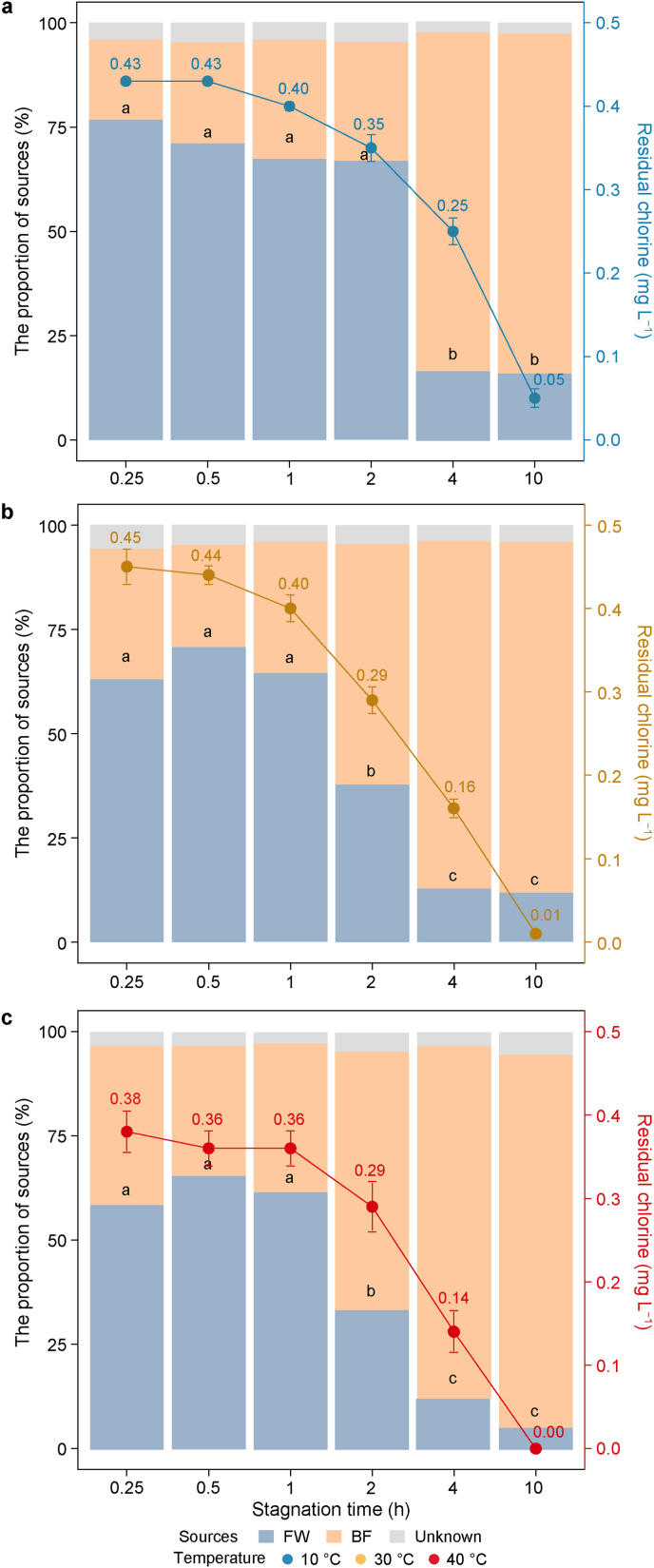
Changes in fresh water (FW) and biofilm (BF) contributions and residual chlorine concentrations: changes over short stagnation up to 10 h at 10 °C (**a**), 30 °C (**b**), and 40 °C (**c**). Points show the concentration of residual chlorine in stagnant water at different times (mean ± standard deviation; right *y*-axis). Different letters indicate the proportional contribution of fresh water to stagnant water microbes that differ significantly (Tukey test, *p* < 0.05).

In general, the contributions from fresh water decreased with the increase in stagnation time, and the decrease rates increased with temperature (Fig. 5; Supplementary Table S5). At 10 °C, within 2 h of stagnation, fresh water was the main contributor to microbes in stagnant water (61.9–83.9 %) (Fig. 5a). With the increase in stagnation time, the contribution of fresh water decreased significantly to 16.6 % at 4 h and 15.8 % at 10 h (*p* < 0.05). When the temperature was increased to 30 and 40 °C, the dominant contribution of fresh water was maintained for only 1 h (64.2 %/59.6 %), dropping significantly to 37.8 %/33.6 % at 2 h, 12.9 %/12.2 % at 4 h, and 11.8 %/5.3 % at 10 h (*p* < 0.05, Fig. 5b and c). The decrease in the contributions from fresh water was accompanied by a continual increase in the contributions of biofilm. When stagnation time reached ≥4 h, BF was the dominant contributor to microbes in stagnant waters at all temperatures (75.5–93.0 %). After 10 h of stagnation, the contributions of fresh water were 15.8 %, 11.8 %, and 5.3 %, while the contributions of biofilm were 81.6 %, 84.2 %, and 89.4 % at 10, 30, and 40 °C, respectively.

## 4 Discussion

Both stagnation time and temperature are critical for microbial water quality at the point of use. In this work, the dynamics of quantity and community of tap water during short daily stagnation at different temperatures were systematically assessed. Our findings provide new insights into the microbiological dynamics of tap water and offer essential knowledge for improving the management of microbial water quality—particularly in the increasingly common sensor-activated faucets that supply warm water.

### 4.1 Turning points of microbial water quality during short stagnation

Residual chlorine declined markedly during short-term stagnation (≤10 h), accompanied by a sharp change in microbial water quality during the short stagnation, measured as an increase in biomass (e.g., ATP, *L. pneumophila*) and a decrease in community diversity (e.g., ASV number, Shannon index). This is consistent with previous observations of microbiological water quality deterioration resulting from water stagnation in building plumbing systems [5,9,29]. However, stagnation research has focused on stagnation times ranging from overnight (8–20 h) to a week (6–7 days) and permanent stagnation [1,6,10]. The present work uncovered the dynamics of microbiological water quality changes during short-term stagnations (0.25–10 h) with high sampling resolution, revealing two distinct turning points at 2 h and 4 h. Water quality remained stable for the first 2 h, then deteriorated sharply between 2 h and 4 h—characterized by increased *L. pneumophila* concentrations and elevated biofilm contributions to bulk water microbes——before stabilizing again beyond 4 h. In practical terms, tap water quality was preserved for up to 2 h, with most deterioration occurring between 2 h and 4 h of stagnation.

This temporal pattern appeared to be strongly associated with residual chlorine decay. When chlorine concentrations fell below effective thresholds, microbial inhibition decreased, promoting activity in both the water and biofilm phases [1]. Consequently, the stagnant water microbiome underwent notable changes, including elevated *L. pneumophila* levels. Simulated experiments were conducted using touchless sensor faucets with water tanks and low-temperature heating (<45 °C), but without hot–cold water mixing valves. Although it is valuable to identify these critical time points, it is important to note that the exact turning points of microbial water quality may vary across different systems. Future research should further investigate the impact of short-term stagnation on microbial water quality dynamics under various conditions (e.g., disinfectant type, touchless sensor faucet system characteristics, supplied water quality, bacterial growth rates, and biofilm release dynamics).

After 4 h of stagnation, microbial communities closely resembled those of tap biofilms (Fig. 3), indicating the dominant contribution of biofilm to the microbes in stagnant water from 4 h onwards—a pattern confirmed by the SourceTracker results (Fig. 5). This This finding aligns with a previous study on shower systems, where community similarity between stagnant water and biofilm increased with stagnation time [14]. Based on this finding, it would be reasonable to assume that the number of observed species would increase because the release of biofilm introduces new microbes into stagnant water [27]. However, the number of observed ASVs in stagnant water decreased sharply, particularly between 2 h and 4 h (Fig. 2), consistent with observations from other shower system studies [13]. This can occur when the number of new species released by biofilms is much lower than the number of extinct species due to stagnation selection. For the microbial ecology of stagnant water, the domination of high-abundance species would exclude a high number of rare species and lead to a decrease in the number of species [39]. This was the case when the stagnation time was 4 h or more, and the microbial communities of all water samples were dominated by *DSSF69* spp. (66.6 ± 9.8 %).

The considerably increased *L. pneumophila* in particular raises infection risks and health concerns for users (Fig. 4). Stagnation in building plumbing systems may promote the growth of *Legionella* spp. and *L. pneumophila*, as has been previously reported [10,11,40]. Consistent with our observations, Huang et al. found a 1.7-fold increase in the infection risk of *L. pneumophila* after 12 h of stagnation [8]. In line with our observations, Huang et al. also reported that infection risk increased rapidly within the first 12 h of stagnation and slowed down afterwards.

In addition to *L. pneumophila*, there might be other opportunistic pathogen risks, such as NTM. Shen et al. reported that NTM concentrations increased nearly 40-fold in shower water after 15 h of stagnation, with 1 in 132 NTMs transferred to indoor air during showers [41]. Marzia et al. reported that 4.1 % of indoor air microbes originate from shower heads, while twice as many (8.8 %) come from tap water [42]. Moreover, previous studies have shown that the emission coefficient for pathogenic microorganisms in water released into the air is higher for faucets (5.6 × 10^−4^ L m^−3^) than for showers (3.4 × 10^−4^ L m^−3^) [[43], [44], [45]]. Therefore, more attention should be given to the potential risks associated with stagnant tap water.

### 4.2 Heating temperature plays a critical role in microbial water quality

When comparing stagnant water with and without heating, it was evident that heating at the tap significantly accelerated residual chlorine decay, which is in line with prior research [25,26]. However, for changes in biomass, opportunistic pathogens, and bacterial community diversity, the increase in temperature showed different impacts. Stagnation at 30 °C exhibited the highest ATP, while 40 °C showed a sharp decrease in ATP at 15 min of stagnation, and both gradually increased until 10 h of stagnation, with the ATP of 40 °C water remaining constantly lower than that of 30 °C.

Most prior research has focused on the impact of heating between 30 and 60 °C and has commonly found a decrease in biomass with an increase in heating temperature [3,28]. Few studies have investigated the application of temperatures below 30 °C. Zhang et al. found that higher temperatures led to a greater increase in biomass during overnight stagnation [27]. It is reasonable to conclude that heating to 30 °C promoted bacterial growth while heating to 40 °C inhibited it, indicating that drinking water microbes might be killed when the water is heated to 40 °C. This is confirmed by the decrease in *Phreatobacter* spp. from 79.9 % to 18.0 % and the 73 % decrease in ATP at 40 °C. The predominance of *Phreatobacter* spp. In chlorinated systems and its low tolerance to high temperatures (>30 °C) have been previously reported in the literature [[46], [47], [48], [49], [50]]. Hence, heating to 40 °C might be a microbiologically safer option than 30 °C.

In contrast, the growth curve of *L. pneumophila* showed its highest growth rate and concentration at 40 °C and its lowest concentration at 30 °C, suggesting that heating to 30 °C might be better than 40 °C for controlling opportunistic pathogens. A previous study found that the optimal growth temperature of *L. pneumophila* ranged from 38 to 42 °C in a constant temperature-controlled glass column simulation experiment [51]. Moreover, the persistence of *L. pneumophila* takes place at temperatures above 50 °C [3], and higher relative abundances exist in hot water (53.6 °C) than in cold water (12.3 °C) [52]. As such, low temperature heating, up to 30 °C, might be a better option than 40 °C for limiting opportunistic pathogens like *L. pneumophila*. This is especially true considering the fact that microbial risks are related to the number of pathogens but not the number of total microbes [53]. If microbes are probiotics, an elevated concentration can be considered beneficial [54]. However, viable *L. pneumophila* and other opportunistic pathogens (e.g., NTM) were not tested in the present work. Further investigations of the growth dynamics of multiple viable opportunistic pathogens under different heating temperatures are needed to provide a more comprehensive understanding of associated infection risks.

Our experimental design draws on previous studies in which the temperature was successively increased by 2.5–7 °C every 5–12 weeks within the same pipe system to explore the influence of temperature (32–58 °C) on the microbial community [3,19,28]. We tested the three temperature conditions sequentially in the same touchless sensor faucet system. However, it is important to note that while temperature significantly affects microbial communities, the differences in microbial community composition observed at different temperatures in this study may also be influenced by biofilm maturity. A lack of temperature independence could bias conclusions about biofilm formation and the dynamics of microbial populations in fresh water, stagnant water, and biofilm phases. To isolate the influence of temperature, the following approaches could be implemented in future research: (1) preparing parallel experimental systems and operating them simultaneously at different temperatures for comparison; (2) resetting the system to allow it to adapt to new temperature conditions; or (3) altering the sequence of temperature testing to further verify the influence of temperature.

For microbial communities during the short stagnation, significant changes occurred at 2–4 h in 10 °C water, and earlier for 30 and 40 °C water, at 1–2 h (Fig. 3), confirming that heating at the tap accelerated microbial water quality changes. This may be explained by the accelerated decay of residual chlorine in heated water, as heat made the residual chlorine drop below critical levels more quickly. In the present work, it was observed that when residual chlorine concentrations were 0.25–0.29 mg L^−1^, the main contributor of microbes in stagnant water shifted from fresh water to biofilm. This agrees with the observations of Ling et al., who found significant changes in cell counts in stagnant water when residual chlorine concentrations were below 0.3 mg L^−1^ [1]. The major contribution from biofilm deserves more attention, as biofilm is a reservoir for opportunistic pathogens and protects microbes from disinfectant [13,[55], [56], [57]]. In this study, it was observed that the residual chlorine dropped below 0.3 mg L^−1^ after 4 h at 10 °C, but after only 2 h at 30 and 40 °C (2 h earlier). This corresponds with the point at which microbial communities showed significant changes. Moreover, the above-mentioned different impacts of 30 and 40 °C temperatures on ATP and *L. pneumophila* were also reflected in microbial community diversity. For example, after half an hour of stagnation, the number of species increased sharply at 40 °C and slightly at 30 °C but decreased at 10 °C.

### 4.3 Practical implications

Continuous hot water supply has become popular in cold regions worldwide, particularly in winter. Electrical touchless sensor faucets with heating functions are usually installed in the sinks of public toilets or home kitchens, with typically short stagnation times of minutes or hours. Our study highlighted the dynamics of microbial water quality changes within 10 h of stagnation at different temperatures. The high-resolution sampling combined with microbial quantity, community, and target opportunistic pathogen analysis suggests that significant deterioration occurs after 4 h (10 °C) and 2 h (30 °C, 40 °C) of stagnation. This means that the microbial water quality guarantee period at the point of use is temperature dependent, and a short flush might be necessary after stagnation of 2–4 h to reduce microbial risks.

Heating temperature emerged as a key control variable. The highest ATP but lowest *L. pneumophila* levels were found at 30 °C, whereas 40 °C produced the lowest ATP but the highest *L. pneumophila* levels. Because touchless sensor faucets lack mixing valves, temperatures above 50 °C—recommended for other hot-water systems such as showers—cannot be achieved. This suggests that an optimal setpoint between 30 °C and 40 °C may minimize both ATP and *L. pneumophila*. To control multiple pathogens simultaneously, a broader temperature range (25–45 °C) warrants investigation.

Given that water heating at the point of use is the third-largest contributor to household energy consumption in the United States, following heating and cooling [58]. Future research on optimal heating temperatures is highly recommended, balancing personal comfort, energy consumption, and microbial safety. This need is underscored by the fact that green buildings designed for resource conservation (e.g., water-saving systems) may inadvertently increase water stagnation in building plumbing systems due to reduced water usage. [59]. Therefore, it is imperative to strengthen studies on water quality changes in short stagnation times.

## 5 Conclusion

In summary, this study investigated the effects of short-term stagnation and temperature on microbial biomass, composition, diversity, and *L. pneumophila* counts in a touchless sensor faucet.

• Residual chlorine concentration and bacterial species richness progressively decreased with prolonged stagnation time, whereas ATP concentrations consistently increased. Heating accelerated chlorine decay, and at 40 °C, the post-stagnation decline in ATP concentration suggested partial microbial inactivation.
• After at least 4 h of stagnation, bacterial communities in stagnant water converged across temperatures, resembling biofilm communities. *DSSF69* spp. dominated in stagnant water (66.6 ± 9.8 %) and was prevalent in biofilms (42.7 ± 12.1 %).
• Heating at the tap accelerated chlorine decay and reduced the time required for biofilm microorganisms to become dominant in stagnant water from 4 h at 10 °C to 2 h at 30 and 40 °C. Such significant changes occurred when residual chlorine dropped below 0.3 mg L^−1^, at which point the dominant members in stagnant water shifted to *DSSF69* spp., a taxon typically enriched in biofilm.
• Heating to 30 °C increased ATP levels while suppressing *L. pneumophila* growth, whereas heating to 40 °C significantly reduced ATP concentrations but promoted *L. pneumophila* proliferation. These results demonstrate that maintaining water heaters at 30 °C is more effective than 40 °C for controlling *L. pneumophila.*

## CRediT authorship contribution statement

**Anran Ren:** Writing – original draft, Visualization, Software, Methodology, Data curation, Conceptualization. **Zihan Dai:** Visualization, Software. **Xiaoming Li:** Writing – review & editing. **Walter van der Meer:** Writing – review & editing. **Joan B. Rose:** Writing – review & editing. **Gang Liu:** Writing – review & editing, Supervision.

## Declaration of competing interest

The authors declare that they have no known competing financial interests or personal relationships that could have appeared to influence the work reported in this paper.
