# Supplementary material for: Temperature-dependent microbial dynamics in touchless sensor faucets during short-term stagnation
Source: Environ Sci Ecotechnol. 2025 Sep 24;28:100624. doi: 10.1016/j.ese.2025.100624 (PMC12513313; doi:10.1016/j.ese.2025.100624)
Supplement: Multimedia component 1 [file mmc1.docx]

Supplementary Information for:

**Temperature-Dependent Microbial Dynamics in Touchless Sensor Faucets During Short-Term Stagnation**

Anran Ren ^a, b, c^, Zihan Dai ^d^, Xiaoming Li ^b, c^, Walter van der Meer ^e, f^, Joan Rose ^g^, Gang Liu ^b, c, h *^

*^a^ Jiangsu Key Laboratory of Industrial Pollution Control and Resource Reuse, School of Environmental Engineering, Xuzhou University of Technology, Xuzhou 221018, China*

*^b^ Key Laboratory of Drinking Water Science and Technology, Research Centre for Eco-Environmental Sciences, Chinese Academy of Sciences, Beijing, 100085, China*

*^c^ University of Chinese Academy of Sciences, Beijing, China*

*^d^ School of Civil and Environmental Engineering, Georgia Institute of Technology, Atlanta, Georgia 30332, USA*

*^e^ Science and Technology, University of Twente, P.O. Box 217, 7500AE Enschede, the Netherlands*

*^f^ Oasen Drinkwater, PO BOX 122, 2800 AC, Gouda, the Netherlands*

*^g^* *Department of Fisheries and Wildlife, Michigan State University, East Lansing, MI 48823, USA*

*^h^ Sanitary engineering, Department of Water management, Faculty of Civil Engineering and Geosciences, Delft University of Technology, P.O. Box 5048, 2600 GA Delft, the Netherlands*

***Corresponding author:**

Prof. Dr Gang Liu,

Research Center for Eco-Environmental Sciences,

Chinese Academy of Sciences,

Beijing, China

email: [gliu@rcees.ac.cn](mailto:gliu@rcees.ac.cn); [g.liu-1@tudelft.nl](mailto:g.liu-1@tudelft.nl)

The supplementary information includes 5 Figures and 5 tables in 13 pages.


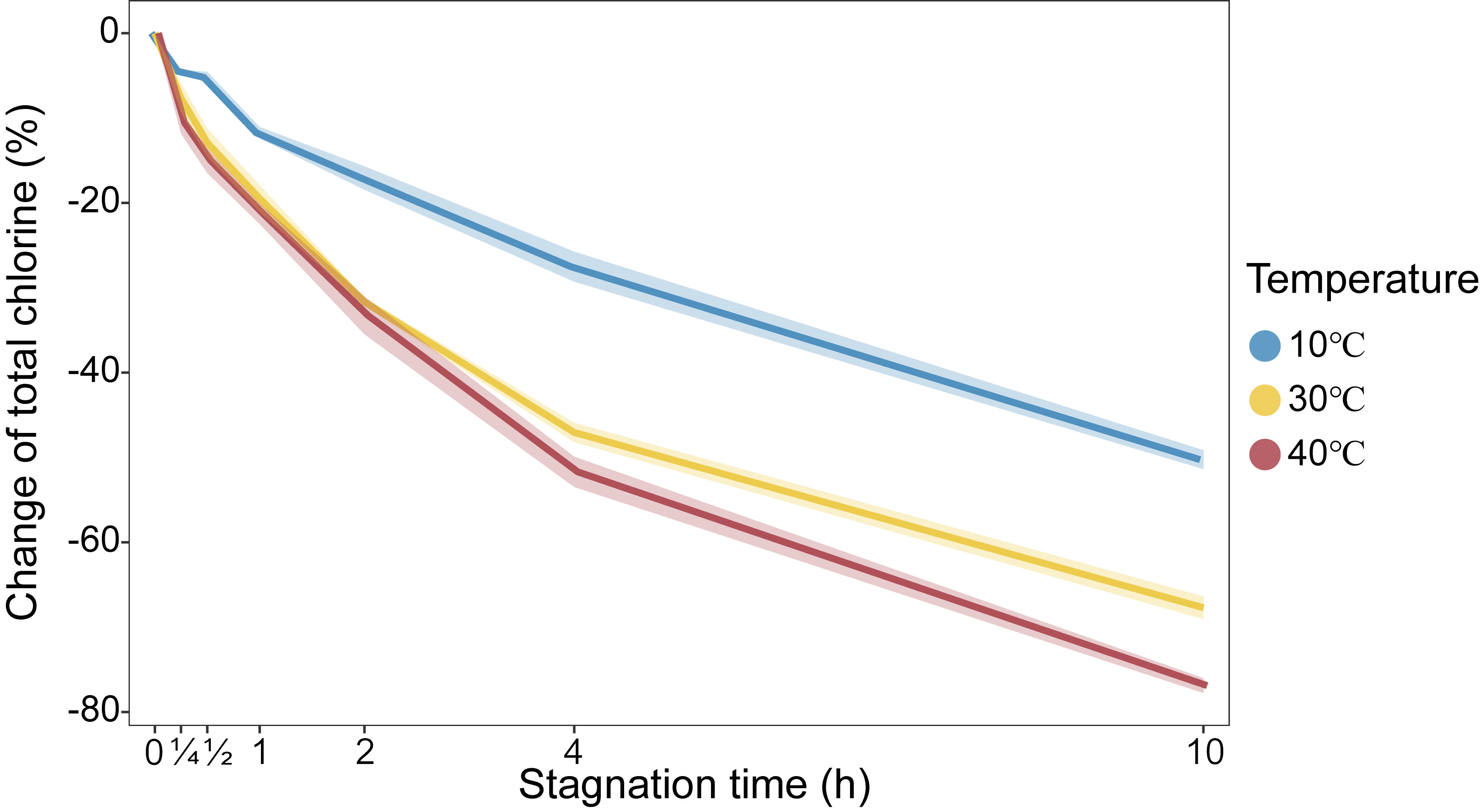


**Figure S1.** *A reaction kinetics model for total chlorine decay in different temperature conditions. Line plots represent mean values with error bands (mean ± standard deviation).*


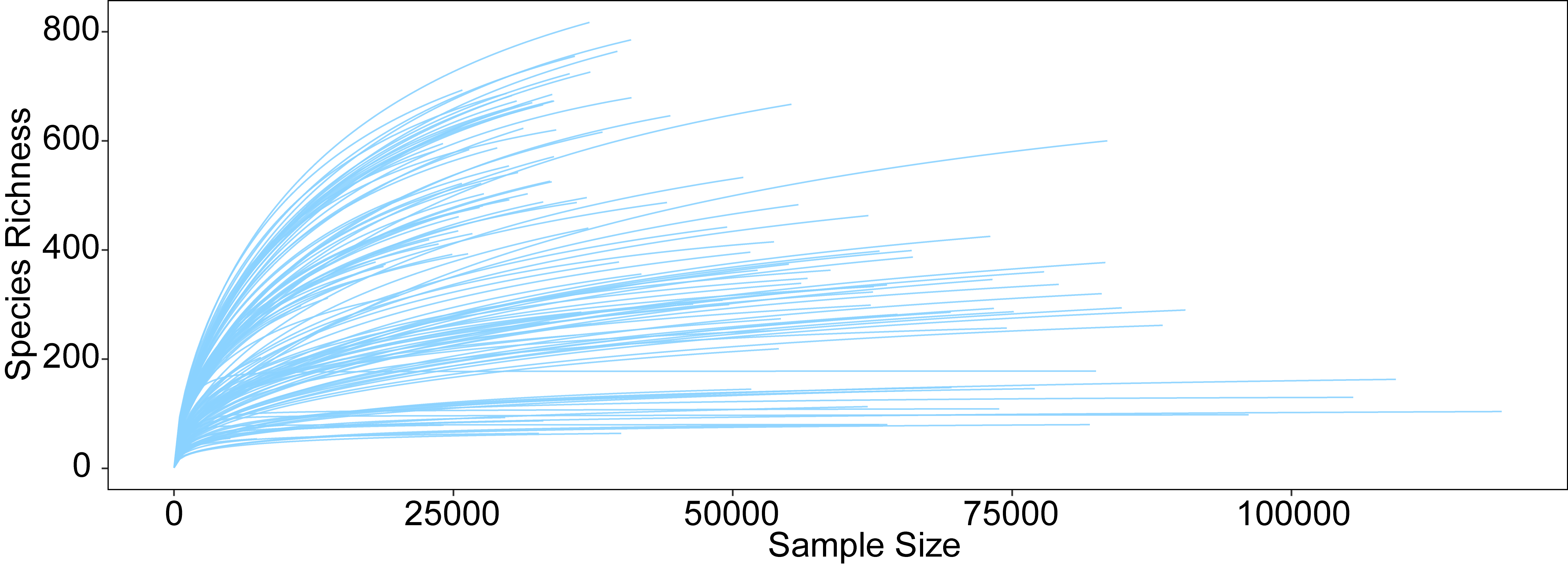


**Figure S2.** *Rarefaction curves of raw sequences.*


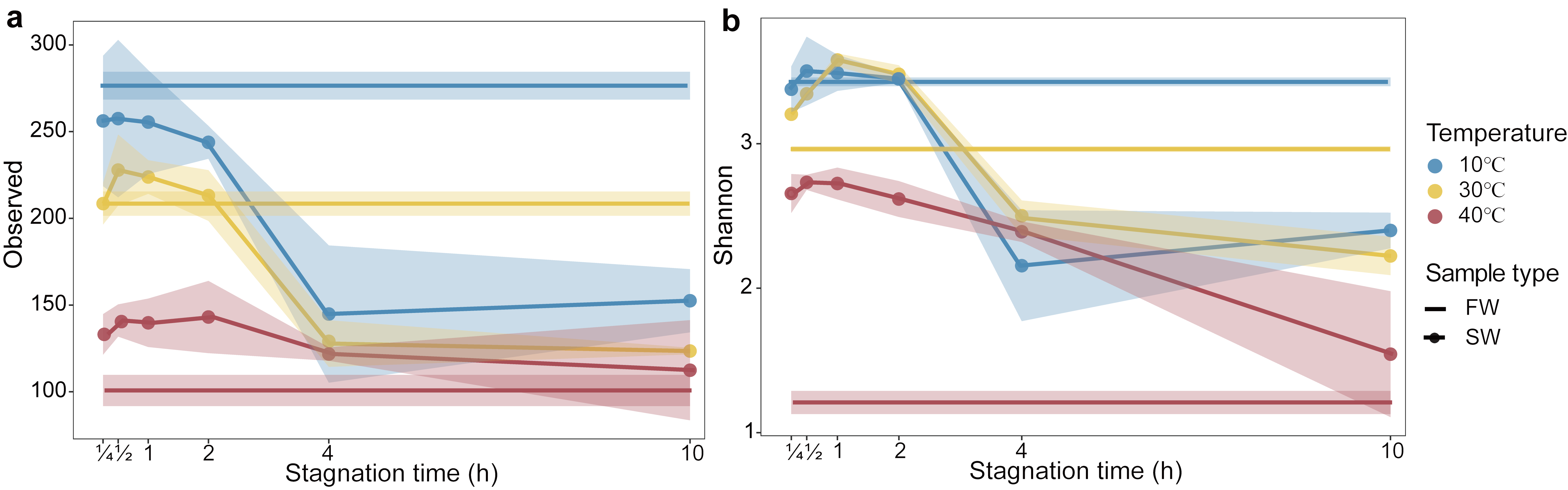


**Figure S3.** *The influences of temperature and stagnation time on the alpha diversity of fresh water (FW) and stagnant water (SW): (A) Observed ASVs and (B) Shannon index.* *Line plots represent mean values with error bands (mean ± s.d.).*

**
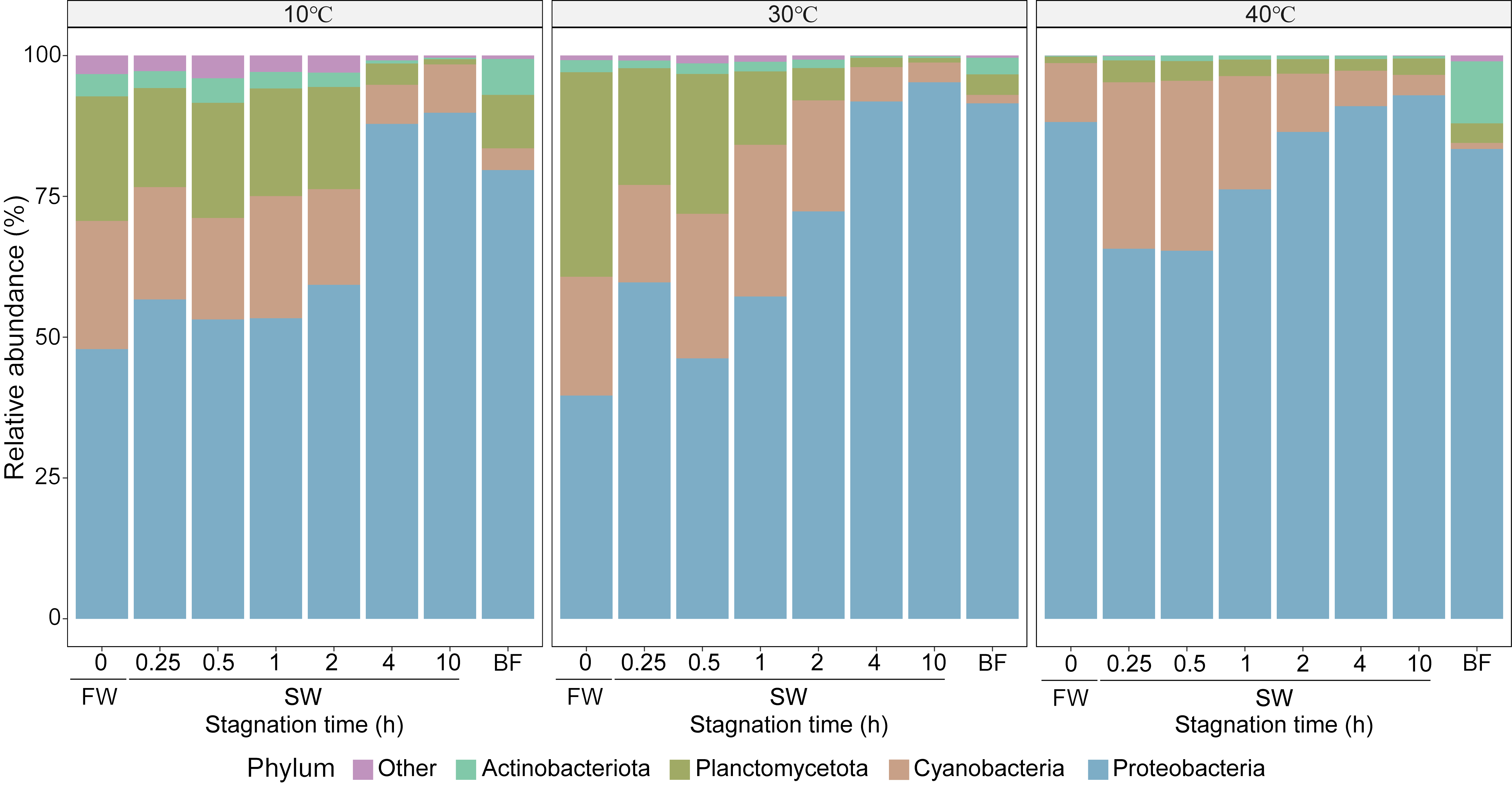
Figure S4.** *The bacterial community composition of fresh water (FW), stagnant water (SW) and biofilm (BF) samples at the phylum level. The* *relative abundance higher than 5% phylum in any individual sample are shown with the remaining phylum as other group.*


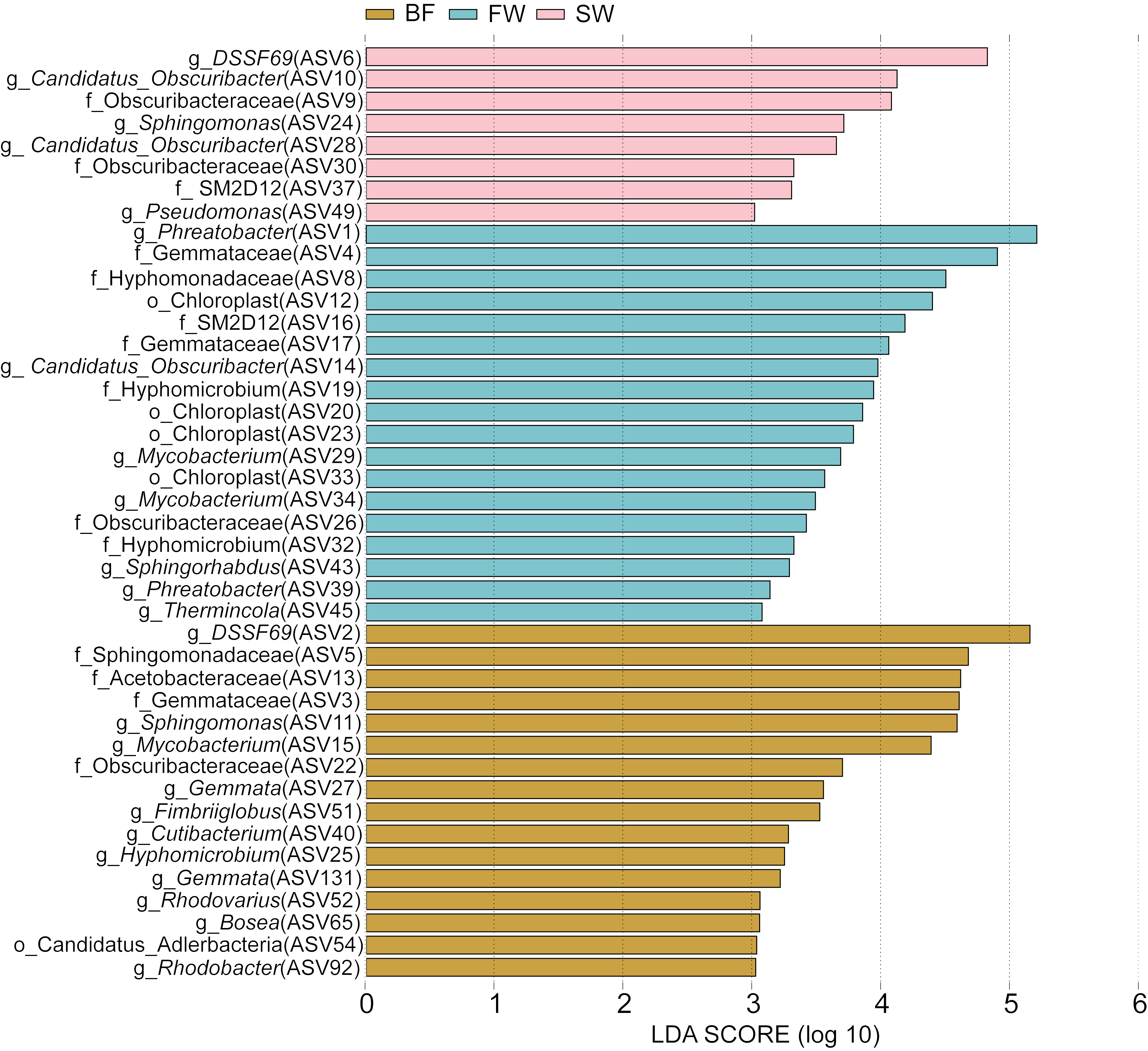


**Figure S5.** *The linear discriminant analysis effect size (LEfSe) selected significantly enriched ASVs in fresh water (FW), stagnant water (SW) and biofilm (BF).*

**Table S1.** *qPCR primers and thermocycling conditions*

| Target | Forward (5’-3’) | Reverse (5’-3’) | Probe | Amplicon size | Time  (Temperature) | Reference |
| --- | --- | --- | --- | --- | --- | --- |
| *Legionella pneumophila* (*mip* gene) | LmipF：AAAGGCATGCAAGACGCTATG | LmipR: GAAACTTGTTAAGAACGTCTTTCATTTG | FAM-TGGCGCTCAATTGCGTTTAACCGA-BHQ1 | 79bp | Denaturation  180S( 95℃)  40 cycles  15S (95℃)  60S (60℃) | [1] |

**Table S2.** *Reference genomic DNA for creation of custom gBlocks Gene Fragments (qPCR standard)*

| Target | Reference genome | NCBI Genomic Sequence | Standard sequence |
| --- | --- | --- | --- |
| *Legionella pneumophila* (*mip* gene) | Macrophage infectivity potentiator Mip [ Legionella pneumophila ] | NZ_CP015941.1 | TAATCCGGAAGCAATGGCTAAAGGCATGCAAGACGCTATGAGTGGCGCTCAATTGGCTTTAACCGAACAGCAAATGAAAGACGTTCTTAACAAGTTTCAGAAAGATTTGATGGCTAA |

**Table S3.** *Summary of real-time quantitative PCR reactions*

| Target | Assay | LOQ, copy number | LOD, number | Amplification efficiency | R^2^ | Slope | Intercept |
| --- | --- | --- | --- | --- | --- | --- | --- |
| *Legionella pneumophila* (*mip* gene) | 1  2  3  4  5 | 10 | 5 | 100.2%  97.4%  96.0%  99.0%  104.1% | 0.994  0.996  0.997  0.996  0.997 | -3.32  -3.39  -3.42  -3.35  -3.23 | 40.52  40.73  41.20  40.62  39.58 |

LOQ, limit of quantification; LOD, limit of detection

**Table S4.** *Taxonomic information of significantly enriched (LDA score >3) ASVs by fresh water (FW), stagnant water (SW) and biofilm (BF).*

| Group | ASV_ID | Kingdom | Phylum | Class | Order | Family | Genus |
| --- | --- | --- | --- | --- | --- | --- | --- |
| FW | ASV1 | Bacteria | Proteobacteria | Alphaproteobacteria | Rhizobiales | Rhizobiales_Incertae_Sedis | *Phreatobacter* |
|  | ASV 4 | Bacteria | Planctomycetota | Planctomycetes | Gemmatales | Gemmataceae | *uncultured* |
|  | ASV8 | Bacteria | Proteobacteria | Alphaproteobacteria | Caulobacterales | Hyphomonadaceae |  |
|  | ASV12 | Bacteria | Cyanobacteria | Cyanobacteriia | Chloroplast |  |  |
|  | ASV16 | Bacteria | Proteobacteria | Alphaproteobacteria | Rickettsiales | SM2D12 |  |
|  | ASV17 | Bacteria | Planctomycetota | Planctomycetes | Gemmatales | Gemmataceae | *uncultured* |
|  | ASV14 | Bacteria | Cyanobacteria | Vampirivibrionia | Obscuribacterales | Obscuribacteraceae | *Candidatus_Obscuribacter* |
|  | ASV19 | Bacteria | Proteobacteria | Alphaproteobacteria | Rhizobiales | Hyphomicrobiaceae | *Hyphomicrobium* |
|  | ASV20 | Bacteria | Cyanobacteria | Cyanobacteriia | Chloroplast |  |  |
|  | ASV23 | Bacteria | Cyanobacteria | Cyanobacteriia | Chloroplast |  |  |
|  | ASV29 | Bacteria | Actinobacteriota | Actinobacteria | Corynebacteriales | Mycobacteriaceae | *Mycobacterium* |
|  | ASV33 | Bacteria | Cyanobacteria | Cyanobacteriia | Chloroplast |  |  |
|  | ASV34 | Bacteria | Actinobacteriota | Actinobacteria | Corynebacteriales | Mycobacteriaceae | *Mycobacterium* |
|  | ASV26 | Bacteria | Cyanobacteria | Vampirivibrionia | Obscuribacterales | Obscuribacteraceae |  |
|  | ASV32 | Bacteria | Proteobacteria | Alphaproteobacteria | Caulobacterales | Hyphomonadaceae |  |
|  | ASV43 | Bacteria | Proteobacteria | Alphaproteobacteria | Sphingomonadales | Sphingomonadaceae | *Sphingorhabdus* |
|  | ASV39 | Bacteria | Proteobacteria | Alphaproteobacteria | Rhizobiales | Rhizobiales_Incertae_Sedis | *Phreatobacter* |
|  | ASV45 | Bacteria | Firmicutes | Clostridia | Thermincolales | Thermincolaceae | *Thermincola* |
| SW | ASV06 | Bacteria | Proteobacteria | Alphaproteobacteria | Sphingomonadales | Sphingomonadaceae | *DSSF69* |
|  | ASV10 | Bacteria | Cyanobacteria | Vampirivibrionia | Obscuribacterales | Obscuribacteraceae | *Candidatus_Obscuribacter* |
|  | ASV09 | Bacteria | Cyanobacteria | Vampirivibrionia | Obscuribacterales | Obscuribacteraceae |  |
|  | ASV4 | Bacteria | Proteobacteria | Alphaproteobacteria | Sphingomonadales | Sphingomonadaceae | *Sphingomonas* |
|  | ASV8 | Bacteria | Cyanobacteria | Vampirivibrionia | Obscuribacterales | Obscuribacteraceae | *Candidatus_Obscuribacter* |
|  | ASV30 | Bacteria | Cyanobacteria | Vampirivibrionia | Obscuribacterales | Obscuribacteraceae |  |
|  | ASV37 | Bacteria | Proteobacteria | Alphaproteobacteria | Rickettsiales | SM2D12 |  |
|  | ASV49 | Bacteria | Proteobacteria | Gammaproteobacteria | Pseudomonadales | Pseudomonadaceae | *Pseudomonas* |
| BF | ASV2 | Bacteria | Proteobacteria | Alphaproteobacteria | Sphingomonadales | Sphingomonadaceae | *DSSF69* |
|  | ASV5 | Bacteria | Proteobacteria | Alphaproteobacteria | Sphingomonadales | Sphingomonadaceae |  |
|  | ASV13 | Bacteria | Proteobacteria | Alphaproteobacteria | Acetobacterales | Acetobacteraceae |  |
|  | ASV3 | Bacteria | Proteobacteria | Alphaproteobacteria | Sphingomonadales | Sphingomonadaceae | *DSSF69* |
|  | ASV11 | Bacteria | Proteobacteria | Alphaproteobacteria | Sphingomonadales | Sphingomonadaceae | *Sphingomonas* |
|  | ASV15 | Bacteria | Actinobacteriota | Actinobacteria | Corynebacteriales | Mycobacteriaceae | *Mycobacterium* |
|  | ASV22 | Bacteria | Cyanobacteria | Vampirivibrionia | Obscuribacterales | Obscuribacteraceae |  |
|  | ASV27 | Bacteria | Planctomycetota | Planctomycetes | Gemmatales | Gemmataceae | *Gemmata* |
|  | ASV51 | Bacteria | Planctomycetota | Planctomycetes | Gemmatales | Gemmataceae | *Fimbriiglobus* |
|  | ASV40 | Bacteria | Actinobacteriota | Actinobacteria | Propionibacteriales | Propionibacteriaceae | *Cutibacterium* |
|  | ASV25 | Bacteria | Proteobacteria | Alphaproteobacteria | Rhizobiales | Hyphomicrobiaceae | *Hyphomicrobium* |
|  | ASV131 | Bacteria | Planctomycetota | Planctomycetes | Gemmatales | Gemmataceae | *Gemmata* |
|  | ASV52 | Bacteria | Proteobacteria | Alphaproteobacteria | Acetobacterales | Acetobacteraceae | *Rhodovarius* |
|  | ASV65 | Bacteria | Proteobacteria | Alphaproteobacteria | Rhizobiales | Beijerinckiaceae | *Bosea* |
|  | ASV54 | Bacteria | Patescibacteria | Parcubacteria | Candidatus_Adlerbacteria |  |  |
|  | ASV92 | Bacteria | Proteobacteria | Alphaproteobacteria | Rhodobacterales | Rhodobacteraceae | *Rhodobacter* |

**Table S5.** *The percentage contributions and standard deviations of the fresh water(FW) and biofilm(BF) for the bacteria in the stagnant water (SW) calculated by SourceTracker, presented as percentage contributions (± standard deviations).*

| Temperature | Sink | Source | | |
| --- | --- | --- | --- | --- |
|  |  | FW | BF | Unknow |
| 10 ℃ | SW (0.25h) | 76.7 ± 5.1% | 19.2 ± 5.8% | 4.1 ± 0.7% |
|  | SW (0.5h) | 71 ± 6.1% | 24.4 ± 7.6% | 4.6 ± 1.7% |
|  | SW(1h) | 67.3 ± 2.5% | 28.7 ± 1.1% | 4.1 ± 1.4% |
|  | SW(2h) | 66.8 ± 3.5% | 28.6 ± 3.8% | 4.6 ± 0.6% |
|  | SW(4h) | 16.6 ± 3.4% | 81.5 ± 4.7% | 1.9 ± 1.3% |
|  | SW (10h) | 15.8 ± 0.6% | 81.6 ± 0.8% | 2.6 ± 0.2% |
| 30 ℃ | SW (0.25h) | 63 ± 2.6% | 31.3 ± 2.7% | 5.7 ± 0.8% |
|  | SW (0.5h) | 70.8 ± 3.5% | 24.5 ± 2.9% | 4.7 ± 0.7% |
|  | SW(1h) | 64.5 ± 7.1% | 31.5 ± 7.1% | 3.9 ± 0.2% |
|  | SW(2h) | 37.8 ± 4.2% | 57.7 ± 4.6% | 4.5 ± 0.4% |
|  | SW(4h) | 12.9 ± 3% | 83.4 ± 3.4% | 3.7 ± 1% |
|  | SW (10h) | 11.8 ± 2.2% | 84.2 ± 3% | 4 ± 0.9% |
| 40 ℃ | SW (0.25h) | 58.6 ± 7.6% | 38 ± 7.1% | 3.4 ± 1.3% |
|  | SW (0.5h) | 65.5 ± 2.6% | 31.3 ± 2.5% | 3.2 ± 0.2% |
|  | SW(1h) | 61.8 ± 5.2% | 35.5 ± 4.8% | 2.7 ± 0.5% |
|  | SW(2h) | 33.6 ± 4.7% | 61.8 ± 3.6% | 4.6 ± 1.2% |
|  | SW(4h) | 12.2 ± 0.7% | 84.5 ± 2.4% | 3.3 ± 0.9% |
|  | SW (10h) | 5.3 ± 1.9% | 89.4 ± 4.6% | 5.4 ± 2.8% |

Reference

[1] H. Wang, M. Edwards, J.O. Falkinham, III, A. Pruden, Molecular Survey of the Occurrence of Legionella spp., Mycobacterium spp., Pseudomonas aeruginosa, and Amoeba Hosts in Two Chloraminated Drinking Water Distribution Systems, Appl Environ Microb. 78 (2012), 6285-6294.
